# SLE-DAS in the First Trimester of Gestation Predicts Maternal Lupus Flares Later in Pregnancy

**DOI:** 10.3389/fphar.2021.660123

**Published:** 2021-04-16

**Authors:** Maddalena Larosa, Nathalie Costedoat-Chalumeau, Gaëlle Guettrot-Imbert, Veronique Le Guern, Nathalie Morel, Diogo Jesus, Luca Iaccarino, Luís Inês, Andrea Doria

**Affiliations:** ^1^ Rheumatology Unit, Department of Medicine, DIMED, University of Padova, Padova, Italy; ^2^ Internal Medicine Department, Hôpital Cochin, Paris, France; ^3^ Centre de Référence Maladies Auto-immunes et Systémiques Rares, Paris, France; ^4^ University of Paris, Paris, France; ^5^ Faculty of Health Sciences, University of Beira Interior, Covilhã, Portugal; ^6^ Rheumatology Department, Centro Hospitalar de Leiria, Leiria, Portugal; ^7^ Rheumatology Department, Centro Hospitalar e Universitário de Coimbra, Coimbra, Portugal

**Keywords:** systemic lupus erythematosus, SLE-DAS, pregnancy, maternal flares, adverse obstetrical outcome

## Abstract

**Introduction:** Systemic Lupus Erythematosus (SLE) mainly occurs during childbearing age. Remission or low disease activity state (LDAS) before conception are recommended by experts to achieve a favourable lupus pregnancy outcome but little is known on the best way to evaluate remission or activity status during pregnancy.

**Objectives:** We tested SLE-disease activity score (SLE-DAS) in the first trimester as predictor of maternal flares and obstetrical complications in 2nd and 3rd trimester in a cohort of SLE pregnant women.

**Patients and Methods:** Inclusion criteria were: 1) women ≥ 18 years; 2) affected with SLE (SLICC 2012); 3) enrolled in two referral centers (Italy and France) 4) with an ongoing singleton pregnancy at 12 weeks (only one pregnancy per patient). Disease activity was assessed at first trimester of pregnancy, using SLE-pregnancy disease activity index (SLEPDAI) and retrospectively applying SLE-DAS. Maternal lupus flares at 2nd and 3rd trimester were defined by the SELENA-SLEDAI Flare Index (SFI). Adverse pregnancy outcome (APO) included: fetal and neonatal death, placental insufficiency with premature delivery <37 weeks, and small for gestational age (SGA) (≤3rd percentile).

**Results:** We included 158 pregnant patients affected with SLE. At first trimester the median SLEPDAI (IQR) was 2 (0–4) and the median SLE-DAS (IQR) 1.32 (0.37–2.08). At least one flare occurred in 25 (15.8%) women during the 2nd and 3rd trimester. APO occurred in 19 (12.0%) patients. A significant correlation between SLE-DAS and SLEPDAI was found in this cohort (Spearman’s *ρ* = 0.97, Figure 1). At multivariate analysis, both SLE-DAS and SLEPDAI predicted maternal flares (adjOR = 1.2; 95% CI = 1.0–1.3, *p* = 0.02; adjOR 1.3, 95% CI = 1.1–1.6 per unit increase, *p* = 0.01, respectively). SLE-DAS and SLEPDAI were associated with APO at univariate analysis (*p* = 0.02).

**Conclusions:** SLE-DAS was highly correlated with SLEPDAI and its use in the first trimester predicted maternal flares in the 2nd and 3rd trimester, making SLE-DAS a reliable instrument to measure SLE activity during pregnancy.

## Introduction

It is well known that Systemic Lupus Erythematosus (SLE) mainly affects women in childbearing age, with a ratio of female-to-male close to 9:1 (Pons-Estel et al., 2017). Therefore, pregnancy is common in SLE patients, who are at risk for complications such as maternal flares, preeclampsia (PE), foetal loss, and preterm birth (Larosa et al., 2019).

**FIGURE 1:**
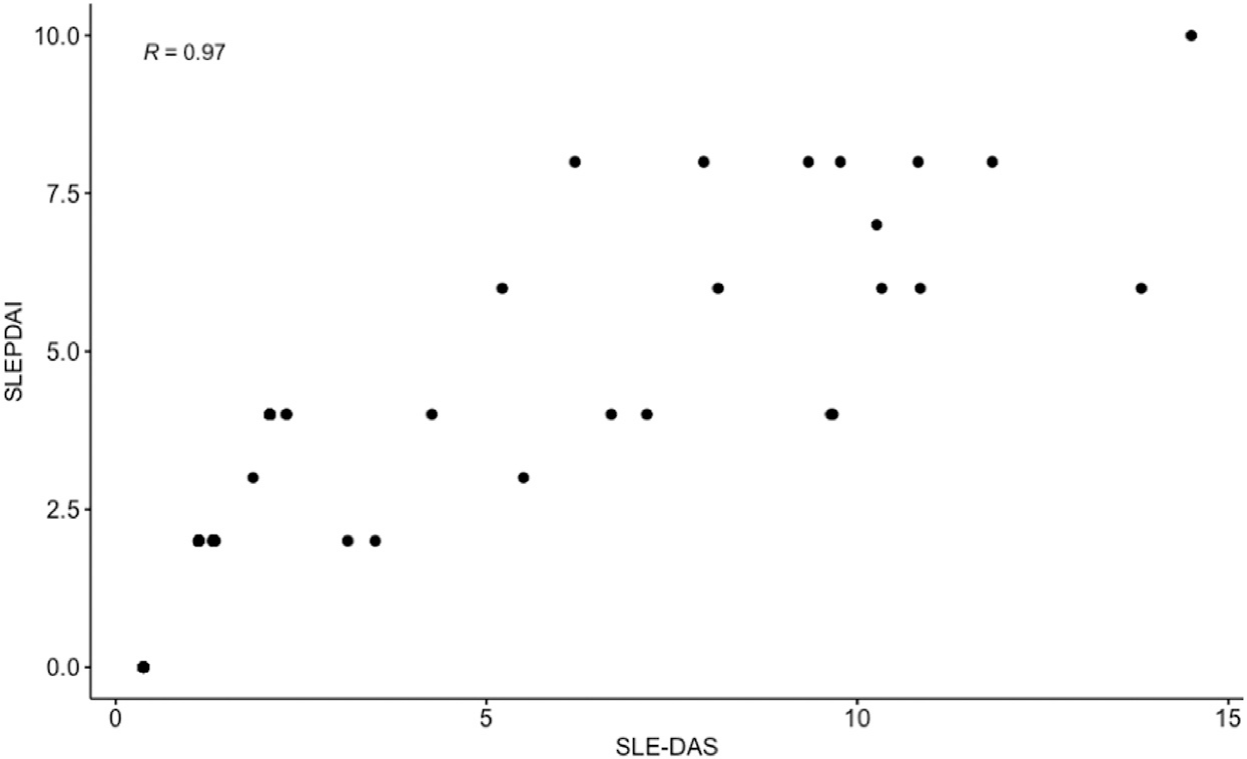
Correlation between SLEPDAI and SLE-DAS at first trimester.

During the last few decades, the outcome of pregnancy in SLE has consistently improved, possibly due to preconception counseling (Andreoli et al., 2017). Risk stratification before conception is pivotal to achieve a favourable outcome (Andreoli et al., 2017) and it should assess SLE remission, autoantibodies (Abs) profile, treatment and previous pregnancy morbidity (Andreoli et al., 2017). Indeed, active/flaring SLE in the 6–12 months before conception is a major risk factor for flares during pregnancy and/or puerperium, as well as for foetal morbidity (i.e. intra-uterine growth restriction-IUGR-, pregnancy loss and preterm delivery) (Andreoli et al., 2017).

In the past decades, efforts has been made to prevent damage accrual in SLE patients by developing treat-to-target (T2T) strategies (Gatto et al., 2019; Jesus et al., 2019a) which primarily pursue the achievement of a durable remission or at least a low disease activity state (LDAS) (Zen et al., 2015; Franklyn et al., 2016; Fischer-Betz and Specker, 2017; Polachek et al., 2017; Ugarte-Gil et al., 2017; van Vollenhoven et al., 2017). Nevertheless, the best definition of such conditions is still under debate and not widely shared.

Recently, Jesus et al. (2019a) developed a new score to measure disease activity, named SLE Disease Activity Score (SLE-DAS) (Jesus et al., 2019a). This score encompasses 17 items and includes some continuous variables (Jesus et al., 2019a). When comparing the performance of this instrument with the SLE disease Activity Index 2000 (SLEDAI-2K) (Gladman et al., 2002), the Authors found that SLE-DAS has a higher accuracy in measuring SLE disease activity, a better sensitivity-to-change and a higher predictive value for damage accrual (Jesus et al., 2019b). Other advantages are that SLE-DAS is a continuous measure of disease activity and includes two important features absent in the SLEDAI-2K, i.e. haemolytic anaemia and lupus enteritis (Jesus et al., 2019a).

During pregnancy, some maternal physiological changes can occur and consequently mislead the evaluation of disease activity. Hence, scoring SLE activity by applying the SLEDAI-2K could be biased. For this reason, Buyon et al. proposed a modification of SLEDAI-2K, which is named SLEPDAI (Buyon et al., 1999). Nonetheless, this score has never been validated.

Since no data on SLE-DAS applicability during pregnancy are available, we aimed to evaluate SLE-DAS in the first trimester as predictor of maternal flares and obstetrical complications.

## Materials and Methods

### Patients

This study was carried out at two referral centers for rare systemic and autoimmune diseases: Rheumatology Unit, University of Padova, Italy (from 2002 to July 2019), and Internal Medicine Department, Cochin Hospital, Paris, France (from 2014 to July 2019, by using all pregnancies from the prospective GR2 study clinicaltrial.gov NCT02450396).

Inclusion criteria were 1) women ≥18 years; 2) affected with SLE (SLICC 2012 criteria) (Petri et al., 2012); 3) with an ongoing singleton pregnancy at 12 weeks (only one pregnancy per patient).

Patients underwent at least one visit in the first trimester, and were followed up according to current clinical practice until the end of pregnancy. This project adheres to the principles of the Declaration of Helsinki and was approved by the local ethics committees.

### Methods

Demographic, clinical and laboratory findings were collected at first trimester by assessing the following variables: age, disease duration (years), skin colour, multiparity, associated anti-phospholipid syndrome (APS) (Miyakis et al., 2006) and SLE manifestations.

Serological and other laboratory data were assessed at first trimester according to standard tests including anti-double stranded DNA (anti-dsDNA), C3 and/or C4 serum levels, and 24-hour (h) proteinuria. Anti-phospholipid (aPL) Abs were also tested including anti-cardiolipin (aCL) IgM and IgG, anti-ß2 glycoprotein-1 (anti-ß2GP1) IgM and IgG, and lupus anticoagulant (LAC) according to standard definitions (Miyakis et al., 2006) and recommendations (Moore, 2014).

Women were considered on therapy when they were taking at least one of the following drugs: hydroxychloroquine (HCQ), prednisone, immunosuppressants (IS), low dose aspirin (LDA) and low molecular weight heparin (LMWH).

### Definition of Disease Activity

Disease activity was assessed at first trimester by applying SLEPDAI (Buyon et al., 1999) and by retrospectively applying SLE-DAS using the online free calculator available at http://sle-das.eu/ (Jesus et al., 2019a).

### Definition of Maternal Flares

Maternal flares in the 2nd and 3rd trimester of gestation were assessed according to SELENA-SLEDAI flare index (SFI) (Petri et al., 2005); flares were subdivided into mild/moderate and severe.

### Definition of Adverse Pregnancy Outcome (APO)

APO were defined with a binary composite score obtained when any of the following event occurred: an otherwise unexplained intrauterine foetal death (IUFD) >12 weeks; a neonatal death within the first 7 days after birth; placental insufficiency (IUGR, PE/eclampsia -E-, Hemolysis, Elevated Liver enzymes, Low Platelets count -HELLP-syndrome, and/or placental abruption) leading to a premature delivery before 37 weeks; small for gestational age (SGA): birth weight ≤3rd percentile according the AUDIPOG curve (Mamelle et al., 2001). Definitions of PE and HELLP are summarized in Supplementary Material.

### Statistical Analysis

We described patients’ characteristics applying mean ± standard deviation (SD) and median with interquartile range (IQR) for parametric and non-parametric continuous variables, respectively. APO’s and maternal flares’ incidences was reported by confidence intervals (CI) set at 95%. The following variables were assessed at first trimester by univariate analysis:

- Continuous: age at pregnancy (years), disease duration (years), SLEPDAI and SLE-DAS scores, prednisone dosage (mg/day);
- Categorical/binary: associated APS, previous renal involvement, serological features including anti-dsDNA, hypocomplementemia (low C3 and/or C4), aPL profile (LAC, IgG/IgM anti-aCL, IgG/IgM anti-beta2GPI, triple positive aPL), active 24 h proteinuria (>0.5 g/day); concomitant treatment including HCQ, prednisone, IS, LDA, and/or LMWH.

Comparison of continuous variables with a parametric and non-parametric distribution was performed using T-test and Wilcoxon’s rank-sum test, respectively. Pearson’s chi-square (or Fisher’s exact test when appropriate) was used to evaluate bivariate associations between categorical variables at univariate analyses. A correlation between SLEPDAI and SLE-DAS in the first trimester was assessed according to Spearman’s correlation test, considering the non-parametric distribution of these scores. Multivariate analysis was performed according to a logistic regression model. The choice of independent variables was based on current knowledge and significant variables at univariate analysis (*p* < 0.1). Two separate multivariate analyses were performed for the outcomes of maternal flares and APO, respectively. Significance at logistic regression analysis was set at 5%. Models’ performance was evaluated using the Hosmer–Lemeshow (HL) goodness-of-fit test and area under the ROC curve (AUC). All statistics were conducted using R software version package 1.3.1073.

## Results

### General Features of SLE Pregnancies at First Trimester

A total of 158 pregnancies in 158 patients affected with SLE were collected (107 enrolled at Internal Medicine Department, Cochin Hospital, and 51 at the Rheumatology Department, University of Padova). Mean ± SD age was 31.9 ± 4.6 years, and 89 (56.3%) women were multiparous.

The majority were White (*n* = 124, 78.5%), followed by Black (*n* = 21, 13.3%), Asian (*n* = 11, 6.9%) and other origin (*n* = 2, 1.3%).

Previous SLE manifestations before pregnancy occurred as following: articular involvement in 125 (79.1%) women, mucocutaneous in 108 (68.4%), renal and haematological in 59 (37.3%) respectively, serositis in 36 (22.8%) and neuropsychiatric (NPSLE) in 7 (4.4%).

Median SLEPDAI (IQR) was 2 (0–4) and median SLE-DAS (IQR) was 1.32 (0.37–2.08).

### General Pregnancy Outcome

We observed 153 (96.8%) live births: their mean birth weight ±SD was 2,946 ± 583 g (2 missing data), and the median delivery term (IQR) was at 38 WG (36–39) (3 missing data). Four (2.5%) IUFD occurred and a medical termination of pregnancy was performed in 1 (0.6%) case.

### Maternal Flares

At least one flare occurred in 25 (15.8%, 95% CI 9.9–20.9) patients during the 2nd and 3rd trimester: 23 (14.6%) women experienced mild/moderate flares and 2 (1.3%) severe renal flares. Overall, we observed that 10 (6.3%) patients had at least one articular flare, 7 (4.4%) mucocutaneous, 5 (3.2%) renal, 3 (1.8%) hematological and serositis flares, respectively. No NPSLE flares occurred in this study (Table 1).

**TABLE 1:** Maternal flares (2nd and 3rd trimester) and APO.

Patients with at least one flare, *n* (%)	25 (15.8)
Articular, *n* (%)	10 (6.3)
Mucocutaneous, *n* (%)	7 (4.4)
Renal, *n* (%)	5 (3.2)
Haematological, *n* (%)	3 (1.9)
Serositis, *n* (%)	3 (1.9)
NPSLE, *n* (%)	0 (0.0)
Patients with at least one APO, *n* (%)	19 (12.0)
IUFD, *n* (%)	4 (2.5)
Preterm delivery due to placental insufficiency, *n* (%)	13 (8.2)
SGA, *n* (%)	3 (2.0)
Neonatal deaths, *n* (%)	0 (0.0)

SLE: Systemic Lupus Erythematosus; NSPLE: Neuropsychiatric SLE; APO: adverse pregnacy outcome; IUFD: intra-uterine fetal death; SGA: small for gestational age.

### APO

Nineteen (12.0%; 95% CI: 7.8–18.0) pregnancies were complicated by obstetrical events (Table 2). Four patients (2.5%) had an IUFD and 13 (8.2%) preterm deliveries due to placental insufficiency. In addition, 3 pregnancies (2.0%, 7 missing data) ended with SGA births. No neonatal deaths occurred.

**TABLE 2:** Univariate analysis: Maternal flares in the 2nd and 3rd trimester. Bold values refer to significative *p* values (*p* < 0.05).

	Overall (*N* = 158)	Flare (*N* = 25)	Non flare (*N* = 133)	*p* Value
Age at pregnancy, mean ± SD (years)	31.9 ± 4.6	31.4 ± 4.6	32.0 ± 4.6	0.50
SLE characteristics				
Associated APS, *n* (%)	18 (11.4)	1 (4.0)	17 (12.8)	0.31
Disease duration, median (IQR)	9 (5–13)	10 (2–13)	9 (5–13)	0.66
Previous renal manifestation, *n* (%)	59 (37.3)	9 (36.0)	50 (37.6)	0.88
Laboratory features in the 1st trimester				
Positive anti-dsDNA, *n* (%)	80 (50.6)	18 (72.0)	62 (46.6)	**0.02**
Low C3/C4, *n* (%)	54 (34.2)	9 (36.0)	45 (33.8)	0.83
24 h-proteinuria > 0.5 g/day, *n* (%)	12 (7.6)	5 (20.0)	7 (5.3)	**0.02**
IgG/IgM anti-cardiolipin, *n* (%)	22 (13.9)	3 (12.0)	19 (14.3)	1.00
IgG/IgM anti-beta2GPI, *n* (%)	12 (7.6)	1 (4.0)	11 (8.3)	0.69
LAC, *n* (%)	24 (15.2)	2 (8.0)	22 (16.5)	0.37
Triple positive aPL, *n* (%)	7 (4.4)	0 (0.0)	7 (5.3)	0.60
Treatment in the 1st trimester				
Prednisone, *n* (%)	72 (45.6)	16 (64.0)	56 (42.1)	**0.04**
Prednisone dose, median (IQR), mg/day	5 (5.0–7.5)	5 (0.0–7.0)	0 (0.0–5.0)	**0.02**
Immunosuppressants, n (%)	44 (27.8)	11 (44.0)	33 (24.8)	0.05
Hydroxychloroquine, *n* (%)	149 (94.3)	23 (92.0)	126 (94.7)	0.63
Low dose aspirin, *n* (%)	88 (55.7)	13 (52.0)	75 (56.4)	0.68
Low molecular weight heparin, *n* (%)	35 (22.6)	3 (12.0)	32 (24.1)	0.29
Disease activity in the 1st trimester				
SLEPDAI, median (IQR)	2 (0–4)	3 (2–6)	2 (0–4)	**0.01**
SLE-DAS, median (IQR)	1.32 (0.37–2.08)	1.85 (1.32–6.19)	1.32 (0.37–2.08)	**0.01**

SD: standard deviation; IQR: interquartile range; SLE: Systemic Lupus Erythematosus; APS: antiphospholipid syndrome; anti-dsDNA: anti-double stranded DNA; 24 h: 24 h; LAC: Lupus anticoagulant; aPL: anti-phospholipid antibodies; SLEPDAI: Systemic Lupus Erythematosus Pregnancy disease Activity Index; SLE-DAS: Systemic Lupus Erythematous disease activity score.

### Univariate Analyses

At univariate analysis, both SLE-DAS and SLEPDAI scores in the first trimester were associated with maternal flares in 2nd and 3rd trimester of gestation (*p* = 0.01 for both). In addition, anti-dsDNA (*p* = 0.02) and active 24 h-proteinuria (*p* = 0.02) were also found to be associated with maternal flares. Regarding treatment, the use of prednisone and prednisone dosage (*p* = 0.04 and *p* = 0.02, respectively) (Table 2). There was a high and significant correlation between SLEPDAI and SLE-DAS (rho = 0.97, *p* < 0.01) in the first trimester.

At univariate analysis, SLE-DAS (*p* = 0.02), SLEPDAI (*p* = 0.02) and anti-dsDNA (*p* = 0.01) were associated with APO (Table 3).

**TABLE 3:** Univariate analysis: Adverse pregnancy outcome (APO). Bold values refer to significative *p* values (*p* < 0.05).

	Overall (*N* = 158)	APO (*N* = 19)	Non-APO (*N* = 139)	*P* Value
Age at pregnancy, mean ± SD (years)	31.9 ± 4.6	31.4 ± 5.2	32.0 ± 4.5	0.57
SLE characteristics				
Associated APS, *n* (%)	18 (11.4)	3 (15.8)	15 (10.8)	0.46
Disease duration, median (IQR)	9 (5–13)	12 (5.5–17)	9 (4.5–13)	0.12
Previous renal manifestation, *n* (%)	59 (37.3)	9 (47.4)	50 (36.0)	0.33
Laboratory features in the 1st trimester				
Positive anti-dsDNA, *n* (%)	80 (50.6)	15 (78.9)	65 (46.8)	**0.01**
Low C3/C4, *n* (%)	54 (34.2)	8 (42.1)	46 (33.1)	0.44
24 h-proteinuria > 0.5 g/day, *n* (%)	12 (7.6)	2 (10.5)	10 (7.2)	0.63
IgG/IgM anti-cardiolipin, *n* (%)	22 (13.9)	3 (15.8)	19 (13.7)	0.73
IgG/IgM anti-beta2GPI, *n* (%)	12 (7.6)	1 (5.3)	11 (7.9)	1.00
LAC, n (%)	24 (15.2)	5 (26.3)	19 (13.7)	0.17
Triple positive aPL, *n* (%)	7 (4.4)	1 (5.3)	6 (4.3)	1.00
Treatment in the 1st trimester				
Prednisone, n (%)	72 (45.6)	11 (57.9)	61 (43.9)	0.25
Prednisone dose, median (IQR), mg/day	5 (5.0–7.5)	5 (0.0–6.8)	0 (0.0–5.0)	0.16
Immunosuppressants, *n* (%)	44 (27.8)	8 (42.1)	36 (25.9)	0.14
Hydroxychloroquine, *n* (%)	149 (94.3)	19 (100.0)	130 (93.5)	0.60
Low dose aspirin, *n* (%)	88 (55.7)	13 (68.4)	75 (53.9)	0.23
Low molecular weight heparin, *n* (%)	35 (22.6)	6 (31.6)	29 (20.9)	0.38
Disease activity in the 1st trimester				
SLEPDAI, median (IQR)	2 (0–4)	2 (2–6)	2 (0–4)	**0.02**
SLE-DAS, median (IQR)	1.32 (0.37–2.08)	1.32 (1.32–2.08)	1.32 (0.37–2.08)	**0.02**

APO: adverse pregnancy outcome; SD: standard deviation; IQR: interquartile range; SLE: Systemic Lupus Erythematosus; APS: antiphospholipid syndrome; anti-dsDNA: anti-double stranded DNA; 24 h: 24 h; LAC: Lupus anticoagulant; aPL: anti-phospholipid antibodies; SLEPDAI: Systemic Lupus Erythematosus Pregnancy disease Activity Index; SLE-DAS: Systemic Lupus Erythematous disease activity score.

### Multivariate Analyses

Regarding maternal flares, since both anti-dsDNA and active 24 h-proteinuria are components of SLEPDAI and SLE-DAS, we did not include these two variables into our logistic regression models in order to avoid collinearity issues. As we found a high correlation between SLEPDAI and SLE-DAS in the first trimester, two different logistic regression models were performed using SLEPDAI and SLE-DAS as explanatory variables (Table 4).

**TABLE 4:** Multivariate logistic regression for maternal flares. Bold values refer to significative *p* values (*p* < 0.05).

		Model 1	Model 2
	Crude OR (95% CI)	AdjOR (95% CI)	AdjOR (95% CI)
SLE-DAS in the first trimester (increase per 1 unit)	1.2 (1.1–1.4)	**1.2 (1.0**–**1.3)**	—
SLEPDAI in the first trimester (increase per 1 unit)	1.4 (1.1–1.7)	—	**1.3 (1.1**–**1.6)**
Prednisone in the first trimester	2.4 (1.0–5.9)	0.9 (0.2–4.4)	0.8 (0.2–3.7)
Prednisone dose (increase per 1 mg/day)	1.1 (1.0–1.3)	1.1 (0.9–1.3)	1.1 (0.9–1.3)
Immunosuppressants in the first trimester	2.4 (1.0–5.8)	1.5 (0.6–4.2)	1.6 (0.6–4.2)
H-L goodness-of-fit^a^		0.90	0.88
AUC curve^b^		0.70	0.69

OR: Odds ratio; adjOR: adjusted OR; CI: Confidence intervals; SLE-DAS: Systemic Lupus Erythematosus disease Activity Score; SLEPDAI: Systemic Lupus Erythematosus Pregnancy disease Activity Index; PDN: prednisone; mg: milligrams; H-L: Hosmer-Lemeshow; AUC: Area under the Curve.

^a^ Refers to p value;

^b^ Refers to AUC.

SLE-DAS in the first trimester was predictor of maternal flares in the 2nd and 3rd trimester (adjusted-adj-OR:1.2; 95% CI:1.0–1.3; *p* = 0.02). Also, SLEPDAI resulted predictor of maternal flares in pregnancy (adjOR = 1.3; 95% CI:1.1–1.6; *p* = 0.01).

We did not perform a multivariate analysis for APO as only anti-dsDNA, SLEPDAI and SLE-DAS resulted statistically associated at univariate analysis; therefore, due to collinearity issues a multivariate analysis was not assessed.

## Discussion

In this study of 158 pregnant women affected with SLE we analyzed disease activity in the 1st trimester assessed by SLEPDAI and SLE-DAS. Although 59 (37.3%) patients had had a previous renal involvement, SLE activity during the 1st trimester was mild: median (IQR) SLEPDAI of 2 (0–4) and median (IQR) SLE-DAS of 1.32 (0.37–2.08). Even though remission/LDAS state cut-off definitions in pregnant patients have not been validated yet, the low scores observed in disease activity indexes in our study, suggest that most patients were not active in the first trimester.

Twenty-five (15.8%) women experienced at least one flare in the 2nd and 3rd trimester. Among them, the majority of our patients had mild/moderate flares (*n* = 23, 14.6%), and only 2 women (1.3%) developed severe flares. This is in line with the PROMISSE study which reported mild/moderate flares occurred in 22.3% and severe flares in 5.5% of SLE patients during 385 prospective pregnancies (Buyon et al., 2015).

APO occurred in 12% of patients, which is also in keeping with the PROMISSE study where these complications occurred in 19% of cases (Buyon et al., 2015). Finally, a high rate of live births was found (96.8%), probably due to the high rate of LDAS/remission state observed in the first trimester of pregnancy in our study. Hence, our results strengthen that remission or LDAS in the early pregnancy is important to avoid fetal complications during pregnancies (Andreoli et al., 2017).

A high correlation between SLE-DAS and SLEPDAI in the 1st trimester was observed in our cohort (*ρ* = 0.97, *p* < 0.01). This is in line with previous findings (Jesus et al., 2019a) who reported a high correlation between the SLE-DAS and SLEDAI-2K. SLEPDAI (Buyon et al., 1999) is a non-validated score adapted to pregnancy that includes the 24 items from the SELENA-SLEDAI with modifications of 15 of them, to avoid wrongly attribution of some physiological or pathological pregnancy changes to SLE (Buyon et al., 1999; Andreoli et al., 2019). Even if SLEDAI-2K and SELENA-SLEDAI are slightly different, correlation between SLE-DAS and both scores was therefore expected.

In multivariate analyses, both SLE-DAS and SLEPDAI at first trimester of pregnancy were independent predictors of SLE flares in the 2nd and 3rd trimester (adjOR, 95% CI = 1.2 (1.0–1.3); adjOR 95% CI = 1.3 (1.1–1.6). In addition, performance of both scores was assessed by AUC and Goodness-of-fit analysis which proved that SLE-DAS model performs slightly better than SLEPDAI model (Table 4). Thus, the high correlation between SLE-DAS and SLEPDAI, the predictive value of SLE-DAS for lupus flares, as well as the aforementioned performance models’ tests prove that SLE-DAS is an appropriate instrument for monitoring SLE disease activity during pregnancy.

Both SLE-DAS and SLEPDAI were associated with APO at univariate analysis (*p* = 0.02), but since anti-dsDNA is already included in both scores, no multivariate analysis could be done. Although our patients had mainly inactive mild SLE, our results suggest that active SLE may still be associated with poor obstetrical prognosis. Such association between disease activity and APO has been already observed in elderly cohorts in which patients had a more active SLE (Clowse et al., 2005). These findings has led to current EULAR recommendations which pinpoint the importance of a stable mild/inactive disease when pregnancy is planned (Andreoli et al., 2017).

Our study has some limitations. First, we applied SLE-DAS retrospectively. This was unavoidable since SLE-DAS was validated in 2019 (Jesus et al., 2019a). Second, in keeping with current recommendations (Andreoli et al., 2017), most pregnancies occurred in patients in clinical remission or LDAS, therefore limiting the possibility to demonstrate a stronger association between high disease activity and APO. The strengths of this study also need to be remarked: first, this a quite large multicentric cohort from 2 referral centers in SLE; second, we acknowledged the role of SLE-DAS as a very simple tool to define disease activity in pregnant patients.

In conclusion, SLE-DAS in the first trimester may be a useful instrument to predict maternal flares in the 2nd and 3rd trimester.

## Data Availability

The raw data supporting the conclusion of this article will be made available by the authors, without undue reservation, to any qualified researcher.
