# Supplementary material for: SLE-DAS in the First Trimester of Gestation Predicts Maternal Lupus Flares Later in Pregnancy
Source: Front Pharmacol. 2021 Apr 16;12:660123. doi: 10.3389/fphar.2021.660123 (PMC8085518; doi:10.3389/fphar.2021.660123)
Supplement: Supplementary file 1 [file datasheet1.docx]

**SUPPLEMENTARY MATERIAL**

**Definition of associated maternal complications.**

1. Preeclampsia (PE) was defined by the presence of systolic blood pressure (BP) ≥ 140 mmHg or a diastolic BP ≥ 90 mmHg, after 20 weeks in a previously normotensive patient with a proteinuria of 0.3 g or higher in a 24-hours urine specimen (20);
2. Haemolysis Elevate d Liver Enzymes Low Platelets (HELLP) syndrome was defined as aspartate amino transferase>2 fold the normal, platelet count <100.000/μL, and lactate dehydrogenase >600 U/L (21).
